# *Formosania tangi*, a new species of suck-loach (Cypriniformes, Gastromyzontidae) from the Jiulongjiang River, southeastern China, with taxonomic notes on *F. fascicauda*

**DOI:** 10.3897/zookeys.1273.184335

**Published:** 2026-03-17

**Authors:** Yang Chen, Jia-Jun Zhou, Jing-Chen Chen, Jin-Quan Yang

**Affiliations:** 1 Shanghai Universities Key Laboratory of Marine Animal Taxonomy and Evolution, Shanghai Ocean University, Shanghai 201306, China Shanghai Universities Key Laboratory of Marine Animal Taxonomy and Evolution, Shanghai Ocean University Shanghai China; 2 Zhejiang Forest Resource Monitoring Center, Hangzhou 310020, China Zhejiang Forest Resource Monitoring Center Hangzhou China; 3 Zhejiang Forestry Survey Planning and Design Company Limited, Hangzhou 310020, China Zhejiang Forestry Survey Planning and Design Company Limited Hangzhou China

**Keywords:** Molecular phylogeny, morphology, new species, taxonomy

## Abstract

For decades, populations of the suck-loach genus *Formosania* from the Jiulongjiang River in Fujian Province, China, have been identified as *F. fascicauda*, a species originally described from in a separate coastal drainage basin in Fuqing County. Employing an integrative taxonomic approach combining morphological and molecular phylogenetic analyses, we demonstrate that the Jiulongjiang River population represents a distinct species, formally described here as *Formosania tangi***sp. nov**. It is diagnosed by a combination of characters: 13 rostral barbels arranged in two rows, a distinct straight, dark, longitudinal stripe along the lateral line, and an emarginate caudal fin. Phylogenetic analyses consistently recover it as a unique evolutionary lineage showing significant genetic divergence from true *F. fascicauda*, with a Kimura 2-parameter distance of 4.16%. The description of *F. tangi* clarifies the taxonomic status of the Jiulongjiang River population and enhances our understanding of species diversity and biogeography of *Formosania* in the coastal drainages of southeastern China.

## Introduction

The suck-loach genus *Crossostoma* was established by Sauvage (1878) based on specimens from the Wuyi Mountain area in Fujian Province, with *C. davidi* designated as the type species. Oshima (1919) independently described the genus *Formosania* from Taiwan, with *F. gilberti* as its type species. Chen (1980) later synonymized *Formosania* under *Crossostoma*, citing the absence of clear morphological distinctions. However, Novák et al. (2006) reinstated *Formosania* as the valid generic name after determining that *Crossostoma* was preoccupied by an earlier gastropod taxon. The genus currently comprises 11 recognized species (Fricke et al. 2026).

*Formosania fascicauda* (Nichols, 1926) was described from Fuqing County, Fujian Province, but the original description (Nichols 1926) omitted the precise collection locality within the county, which is drained by both the Longjiang (龙江) and Yuxi rivers (渔溪). Fang (1934) examined a cotype and noted the diagnostic features distinguishing it from *F. lacustris* (Steindachner, 1908) and *F. stigmata* (Nichols, 1926). Later, Chen (1980) identified specimens from the Jiulongjiang (九龙江) and Jinjiang rivers (晋江) as *F. fascicauda* but did not compare these specimens to topotypic material from Fuqing. Consequently, subsequent researchers have consistently assigned the Jiulongjiang River population to *F. fascicauda* without verification (Zhang and Zhao 2016; Sun et al. 2023).

To evaluate the taxonomic status of the Jiulongjiang River population and its relationship to *F. fascicauda*, we first ascertained the precise type locality of *F. fascicauda*. The key holotype data and photographs for this study were provided by the Department of Ichthyology, American Museum of Natural History. Comprehensive specimens were then collected from multiple coastal drainages across Fujian Province, including the Jiulongjiang River system. Integrated morphological comparisons and molecular phylogenetic analyses demonstrate that the Jiulongjiang River population is morphologically and genetically distinct from true *F. fascicauda*, supporting its formal recognition as a new species, which we describe herein.

## Materials and methods

### Specimen collection, processing, and measurement

**Figure 1.**
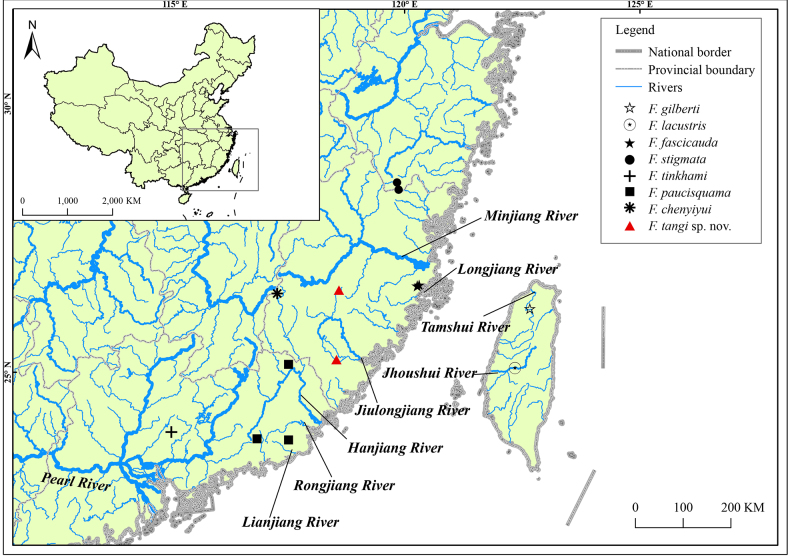
Collection sites of *Formosania* species for morphological comparison (data for *F. lacustris* and *F. gilberti* from Yeh et al. 2024).

Details of specimen collection are provided in Table 1 and Fig. 1. Fish were collected using hand nets. Following capture, specimens were immediately euthanized by immersion in a solution of methanesulfonate (MS-222). Those designated for molecular analysis were fixed directly in 95% ethanol. Specimens intended for morphological study were initially fixed in a 10% formalin solution for approximately 48 hours before being transferred to 65% ethanol for long-term preservation. All collected specimens are deposited in the Fish Museum of Shanghai Ocean University (**SHOU**). Comparative materials are provided at the end of this article.

**Table 1.** The samples used in molecular analysis with their localities, voucher information and GenBank accession.

Species	Specimen voucher	Sampling localities	River system	GenBank accession	Source
*F. tangi* sp. nov.	SHOU20240801010-14	Datian County, Fujian	Jiulong-jiang	PX146867–PX146871	This study
*F. fascicauda*	SHOU20241122001-04	Fuqing City, Fujian	Long-jiang	PX146873–PX146876	This study
	SHOU20241019041	Fuqing City, Fujian	Yu-xi	PX146872	This study
*F. davidi*	SHOU202106251-53, 62-63	Qingyuan County, Zhejiang	Min-jiang	OQ605818–OQ605822	Sun et al. 2023
*F. fasciolata*	SHOU202107001-05	Taishun County, Zhejiang	Feiyun-jiang	OQ605808–OQ605812	Sun et al. 2023
*F. galericula*	SHOU202106273, 75-77, 93	Qingyuan County, Zhejiang	Ou-jiang	OQ605803–OQ605807	Sun et al. 2023
*F. immaculata*	SHOU202106312-16	Wuyi County, Zhejiang	Ou-jiang	OQ605813–OQ605817	Sun et al. 2023
*F. stigmata*	SHOU202201013, 19, 27	Yanping County, Fujian	Min-jiang	OQ605800–OQ605802	Sun et al. 2023
*F. paucisquama*	SHOU202110011	Puning County, Guangdong	Lian-jiang	OQ605798	Sun et al. 2023
	SHOU202110028	Jiexi County, Guangdong	Rong-jiang	OQ605799	Sun et al. 2023
*F. tinkhami*	SHOU202110086	Longmen County, Guangdong	Zhu-jiang	OQ605795	Sun et al. 2023
*Vanmanenia stenosoma*	–	Shaoxing City, Zhejiang	Qiantang-jiang	MZ853160	GenBank
*F. chenyiyui*	SHOU20150001	Changting County, Fujian	Han-jiang	OQ605797	Sun et al. 2023
*F. lacustris*	–	Taiwan	–	AY392454–AY392461, AY392463–AY392467	GenBank
	–	Taiwan	–	KX056126	GenBank
	–	Taiwan	–	NC001727	GenBank

Morphometric measurements followed Sun et al. (2025) and were obtained point-to-point with digital callipers (0.01 mm) for all mainland Chinese *Formosania* species with two rostral barbel rows and 13 rostral barbels, including *F. fascicauda*, *F. stigmata*, *F. paucisquama* (Zheng, 1981), *F. tinkhami* (Herre, 1934), and the Jiulong River population; all values are presented as percentages of standard or head lengths. Meristics were counted under a dissecting microscope, primarily on the left side. Abbreviations used in the text: standard length (**SL**), from tip of the snout to the last half-centrum; total length (**TL**), from tip of the snout to the most posterior of the caudal fin; head length (**HL**), from tip of the snout to the most posterior point of the operculum. For *F. lacustris* and *F. gilberti*, only meristic data were cited from Yeh et al. (2024). The meristic data from this source are recalculated and verified by us, superseding the originally published values.

### DNA extraction, PCR amplification, and sequencing

To clarify the genetic relationship between the Jiulongjiang River population and the topotypic *F. fascicauda* population from Fuqing, 10 individuals from the Jiulongjiang, Longjiang, and Yuxi rivers were selected for amplification and sequencing of the mitochondrial cytochrome *b* (*Cytb*) gene. Genomic DNA was extracted from clips of the right pectoral fin from ethanol-preserved specimens. Samples were systematically labelled according to their collection dates.

The mitochondrial cytochrome *b* gene was amplified via polymerase chain reaction (PCR). The 25 μL PCR reaction mixture consisted of 9.5 μL ddH_2_O, 1 μL of each primer (10 μM), 1 μL template DNA, and 12.5 μL of 2× Taq PCR Master Mix (Sangon Biotech Co., Ltd, Shanghai, China). The thermocycling protocol was as follows: initial denaturation at 95 °C for 3 min; 35 cycles of denaturation at 94 °C for 30 s, annealing at 54 °C for 45 s, and extension at 72 °C for 1 min. The primers used for both amplification and sequencing were QcytbL (5'-GACTTGAAGAACCACCGTTGTTATT-3') and QcytbH (5'-TCTTCGGATTACAAGACCGAT-GCTTT-3') (Chen et al. 2024). PCR products were purified and sequenced commercially by Sangon Biotech (Shanghai, China). Sequence chromatograms were assembled and edited using SeqMan module of DNASTAR Lasergene (Burland 2000). The newly generated sequences have been deposited in the GenBank database, with accession numbers provided in Table 1.

### Phylogenetic reconstruction

A dataset for phylogenetic analysis was assembled, comprising the *Cytb* sequences generated in this study along with homologous sequences for other *Formosania* species downloaded from the NCBI database. No *Cytb* sequence is publicly available under the name *F. gilberti*, which has been historically treated as a junior synonym of *F. lacustris* – the name under which all previous genetic data are deposited. Although Yeh et al. (2024) revised *F. gilberti* as a valid species, the specific sequence data are yet not available in the NCBI database. Given that their work confirms its close relationship to *F. lacustris*, we conclude that its exclusion does not compromise our phylogenetic results, which focus on the distinct species of mainland China. A sequence of *Vanmanenia stenosoma* (Boulenger, 1901) (GenBank accession MZ853160) was downloaded and included as the outgroup. The final aligned dataset contained 53 sequences.

Phylogenetic analyses were conducted using PHYLOSUITE (Zhang et al. 2020). Sequence alignment was performed with MAFFT (Katoh and Standley 2013) under the automatic strategy and normal alignment mode. The best-fit nucleotide substitution models for maximum-likelihood (ML) and Bayesian-inference (BI) analyses were selected using ModelFinder (Kalyaanamoorthy et al. 2017) based on the Bayesian Information Criterion (BIC). The ML tree was constructed with IQ-TREE (Nguyen et al. 2015) using the TIM2+F+I+G4 model, with all other parameters set to default. The BI tree was constructed using MrBayes v. 3.2.6 (Ronquist et al. 2012) with the GTR+F+I+G4 model, running two parallel Markov Chain Monte Carlo (MCMC) analyses for 1 million generations, sampling every 100 generations. The MCMC analysis was considered to have converged, as the average standard deviation of split frequencies dropped below 0.01 (final value = 0.003854). The first 25% of trees were discarded as burn-in. Resulting phylogenetic trees were visualized and annotated using FigTree v. 1.4.4 (Rambaut 2018). Genetic distances were calculated using MEGA11 (Tamura et al. 2021) with the Kimura 2-parameter model (rates among sites: G4), computing average interspecific genetic distances.

## Results

### Taxonomic account

**Family Gastromyzontidae Hora, 1950**

**Genus *Formosania* Oshima, 1919**

#### Formosania tangi sp. nov.

Taxon classificationAnimaliaCypriniformesBalitoridae

6E9A8248-8666-5E86-A484-4A95CB0E4CD2

https://zoobank.org/39BFBF89-6178-4A97-AECB-72345DC20A10

**Figure 2.**
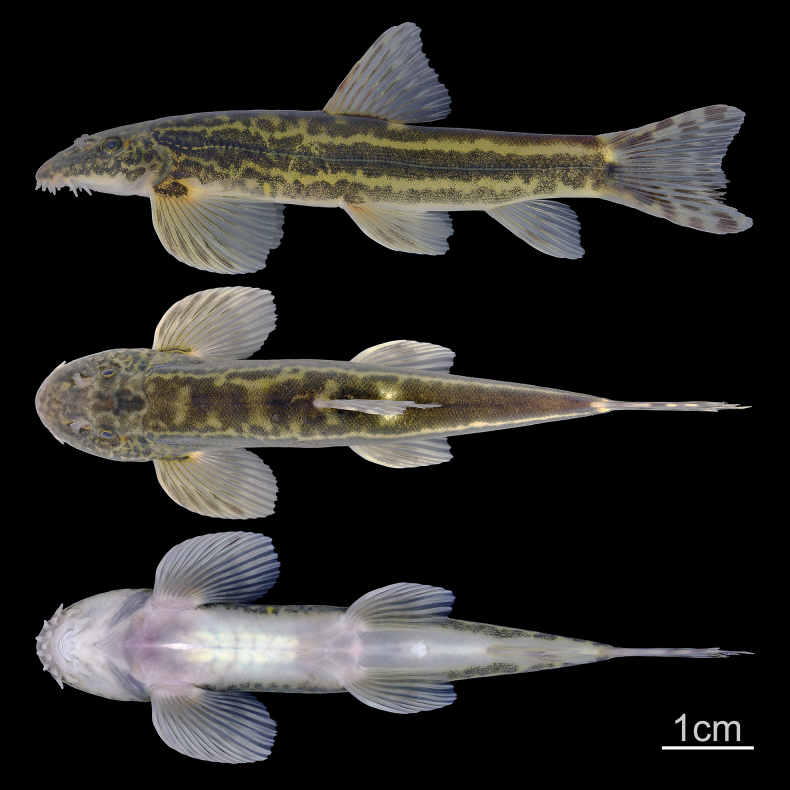
Lateral, dorsal, and ventral views of *Formosania tangi* sp. nov., holotype, adult, SHOU20251010601, 63.74 mm SL.

**Figure 3.**
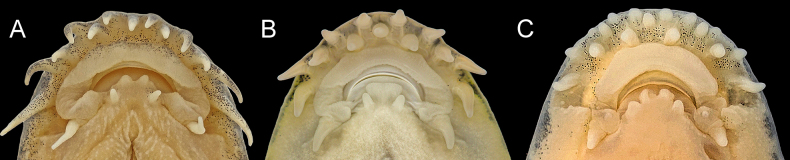
Ventral view of the head, showing the arrangement of rostral barbels. **A**. One-row type in *Formosania davidi* (SHOU20230717003, from Minjiang River, Wuyishan City, Fujian Province); **B**. Two-row type in *F. tangi* sp. nov. (SHOU20251010601, holotype); **C**. *F. chenyiyui* (SHOU20250402004, from Tingjiang River, Changting County, Fujian Province).

**Figure 4.**
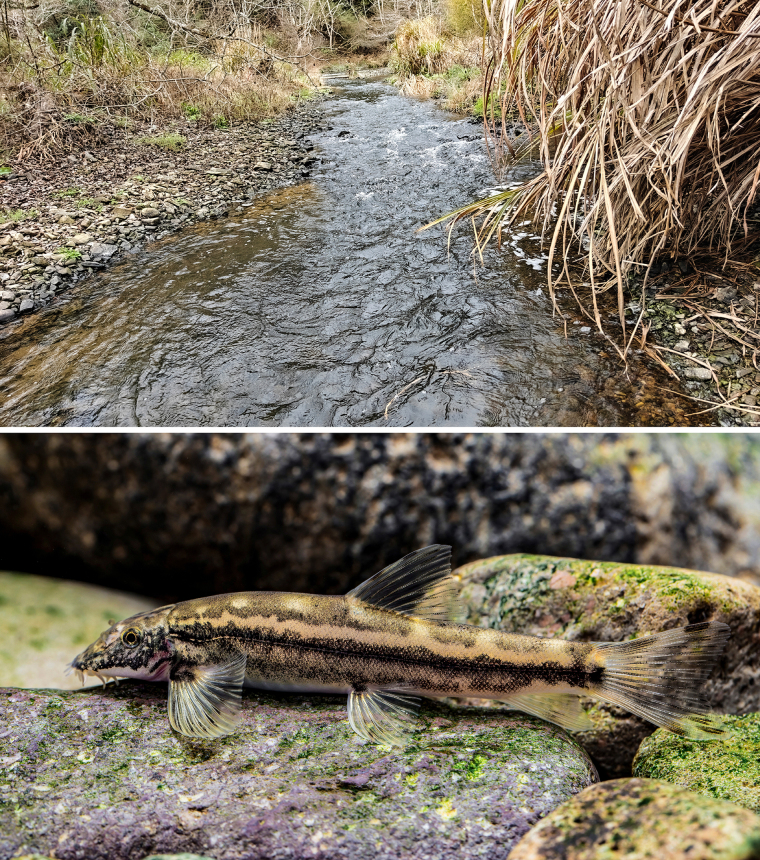
Habitat (photographed by Yong-Sheng Lin) and live appearance of *Formosania tangi* sp. nov. (photographed by Hao-Jun Chen).

Figs 2, 3B, 4, 7D, 8A

Crossostoma fascicauda : Chen 1980: 104 (partim: Jiulongjiang River in Longyan, Fujian).
Crossostoma fascicauda : Chen and Tang in Le 2000: 438–567 (partim: Jiulongjiang River in Longyan, Fujian).
Formosania fascicauda : Sun et al. 2023: 207–221. (JiulongJiang River in Nanjing, Fujian).

##### Type material.

***Holotype*** • SHOU20251010601, TL 79.13 mm, SL 63.74 mm; China, Fujian Province, Sanming City, Datian County, Taoyuan Town, Jiulongjiang River; 25.843°N, 117.577°E, elevation 801 m; Yong-Sheng Lin leg.; 10 Oct. 2025 (Fig. 2). ***Paratypes*** • SHOU20251010602, SL 56.76 mm; collected from the type locality; Yong-Sheng Lin leg.; 10 Oct. 2025. • SHOU20240704603 to -605, 4 specimens, SL 56.76–73.69 mm; collected from the type locality; 4 July 2024. • SHOU202201083 to SHOU202201093, 11 specimens, SL 50.53–81.06 mm; China, Fujian Province, Zhangzhou City, Nanjing County, Jiulongjiang River; 24.630°N, 117.083°E, elevation 766 m; Yang Chen & Jia-Jun Zhou leg.; January 2022.

##### Diagnosis.

The new species can be distinguished from congeners by combination of following characters: rostral barbels 13, well developed, arranged in two rows (Fig. 3B) (vs one row in *F. davidi*, *F. fasciolata*, *F. galericula*, and *F. immaculata* – Fig. 3A; 12–15 rudimentary barbels in *F. chenyiyui* – Fig. 3C); lateral line accompanied by broad, dark, longitudinal band, margins weakly undulating or nearly straight, pale stripe between lateral line band and dark dorsal surfaces generally continuous straight (vs a thin, black, longitudinal line with alternating patches, pale stripe intermittent wavy or absent in *F. fascicauda*, *F. stigmata*, and *F. paucisquama*; lateral line with alternating small dark dots, flanks with interrupted, short, dark streaks or small patches along dorsal and ventral margins in *F. tinkhami*; lateral line with alternating small, dark dots, flanks vermicular in *F. gilberti*; flanks unblotched, with lateral streak in *F. lacustris*); fully expanded caudal-fin emarginate (Fig. 7D) (vs subtruncate in *F. fascicauda* (Fig. 7B), *F. davidi*, *F. fasciolata*, *F. galericula*, and *F. immaculata* (Fig. 7A)).

##### Description.

Dorsal iii-8, anal ii-5, pectoral i-14-15, pelvic i-8. Lateral-line canal pores and scales 72–88. Morphometric measurements are given in Table 2. See Fig. 2 for lateral, dorsal, and ventral views of the body.

**Table 2.** Statistical analysis of morphometric data for two-row barbel *Formosania* species.

Characters	*F. tangi* sp. nov. (*N* = 16)			*F. fascicauda* (*N* = 16)		*F. stigmata* (*N* = 14)		*F. paucisquama* (*N* = 19)		*F. tinkhami* (*N* = 6)	
	Holotype	Range	Mean+SD	Range	Mean+SD	Range	Mean+SD	Range	Mean+SD	Range	Mean+SD
Standard length (mm)	63.74	50.53–81.06	62.53 ± 9.16	53.98–83.30	64.08 ± 9.46	49.95–85.47	62.05 ± 11.07	49.04–65.76	56.31 ± 5.33	51.98–80.29	66.63 ± 11.38
**% of standard length (SL)**											
Body depth (BD)	16.36	13.23–17.32	**15.92 ± 1.11^b^**	15.77–19.24	17.01 ± 0.78^ab^	16.10–21.93	**18.28 ± 1.89^a^**	14.14–19.40	17.10 ± 1.57^ab^	14.93–16.69	**15.99 ± 0.67^b^**
Head length (HL)	27.09	19.95–27.09	**22.05 ± 1.95^b^**	21.37–25.08	**23.60 ± 1.05^a^**	23.59–26.66	**24.72 ± 0.88^a^**	22.65–27.37	24.67 ± 1.39^ab^	23.26–24.11	23.59 ± 0.33^ab^
Dorsal head length (DHL)	22.26	20.34–24.06	**22.64 ± 1.00^b^**	21.63–24.07	**22.61 ± 0.62^b^**	22.32–26.24	**24.08 ± 0.96^a^**	21.36–26.41	**24.53 ± 1.33^a^**	22.70–24.72	23.69 ± 0.86^ab^
Head depth (HD)	11.31	10.50–13.29	11.71 ± 0.74	10.87–12.79	11.73 ± 0.52	10.65–13.51	11.78 ± 0.72	10.89–13.69	11.95 ± 0.67	11.31–12.31	11.78 ± 0.36
Head width (HW)	18.73	17.64–20.45	**18.89 ± 0.77^b^**	17.28–20.35	**18.61 ± 0.80^b^**	18.01–21.16	19.33 ± 1.07^ab^	18.00–22.69	**20.24 ± 1.29^a^**	18.39–20.12	19.30 ± 0.77^ab^
Caudal-peduncle length (CPL)	15.03	7.87–15.03	**11.07 ± 2.19^b^**	10.70–15.07	**12.76 ± 0.99^a^**	10.42–15.01	**12.81 ± 1.35^a^**	11.30–14.73	**12.82 ± 1.02^a^**	10.67–12.14	11.42 ± 0.60^ab^
Caudal-peduncle depth (CPD)	10.68	9.25–11.47	**10.28 ± 0.59^b^**	10.33–12.62	**11.33 ± 0.70^a^**	9.74–11.99	**10.54 ± 0.65^b^**	9.23–12.07	10.78 ± 0.86^ab^	9.45–10.96	**10.15 ± 0.52^b^**
Dorsal-fin length (DFL)	22.48	20.28–24.36	**22.32 ± 1.05^b^**	19.96–23.28	**21.28 ± 0.99^b^**	20.76–24.96	**22.44 ± 1.38^b^**	21.06–26.94	**24.27 ± 1.58^a^**	22.12–24.68	23.63 ± 0.90^ab^
Pectoral-fin length (PFL)	24.24	21.77–26.42	**24.03 ± 1.15^b^**	22.98–26.70	**24.76 ± 1.16^b^**	21.46–27.40	**25.47 ± 1.67^b^**	22.87–29.74	**27.25 ± 1.74^a^**	24.18–26.50	25.39 ± 1.10^ab^
Pelvic-fin length (VFL)	20.80	18.80–21.07	**19.98 ± 0.86^b^**	18.47–22.06	**20.50 ± 0.93^b^**	18.03–22.38	**20.52 ± 1.44^b^**	19.97–24.08	**22.33 ± 1.24^a^**	19.73–21.97	20.87 ± 0.94^ab^
Anal-fin length (AFL)	18.86	16.96–19.73	**18.57 ± 0.73^b^**	16.85–19.77	**18.19 ± 0.79^b^**	17.42–20.76	**18.71 ± 0.98^b^**	17.43–25.80	**20.39 ± 1.99^a^**	17.59–19.89	18.83 ± 0.85^ab^
Dorsal-fin base length (DBL)	13.07	10.85–14.15	**12.73 ± 1.09^b^**	12.32–15.75	**14.23 ± 0.71^a^**	12.27–17.56	**14.57 ± 1.29^a^**	12.17–17.50	**14.28 ± 1.42^a^**	12.59–15.18	13.69 ± 0.99^ab^
Pectoral-fin base length (PBL)	7.66	7.40–8.57	**8.08 ± 0.39^c^**	7.81–9.97	**8.75 ± 0.53^b^**	8.59–9.59	**9.14 ± 0.28^ab^**	8.63–10.18	**9.50 ± 0.47^a^**	8.73–10.64	**9.58 ± 0.63^a^**
Pelvic-fin base length (VBL)	5.04	3.90–5.47	**4.94 ± 0.39^b^**	5.11–7.28	**5.79 ± 0.60^a^**	5.46–6.64	**5.93 ± 0.40^a^**	4.70–6.14	**5.54 ± 0.45^a^**	5.28–5.97	**5.63 ± 0.31^a^**
Anal-fin base length (ABL)	7.56	6.43–8.43	7.49 ± 0.64	6.55–9.11	8.05 ± 0.76	6.87–10.58	8.27 ± 0.99	6.37–8.99	7.76 ± 0.74	6.88–8.98	8.04 ± 0.70
Pre-dorsal length (PDL)	50.16	49.30–53.10	**50.88 ± 1.17^c^**	48.33–53.34	**49.93 ± 1.32^c^**	50.67–57.69	**53.71 ± 1.93^a^**	49.96–56.01	**52.40 ± 1.60^ab^**	49.51–51.73	**50.71 ± 0.82^bc^**
Prepectoral length (PPL)	21.71	18.90–23.21	20.79 ± 1.00 ^ab^	18.96–21.62	**19.94 ± 0.71^b^**	18.31–22.56	20.59 ± 1.05 ^ab^	19.17–23.82	**21.30 ± 1.23^a^**	19.79–20.80	20.41 ± 0.41^ab^
Pre-pelvic length (PVL)	53.09	51.26–55.61	**52.97 ± 0.93^a^**	50.07–53.41	**51.46 ± 1.06^b^**	48.92–52.56	**51.06 ± 1.18^b^**	49.69–54.58	51.98 ± 1.26^ab^	51.26–53.93	52.48 ± 0.89^ab^
Pre-anal-pore length (PAPL)	67.95	66.47–72.42	68.52 ± 1.46	66.07–70.50	68.06 ± 1.16	64.93–69.69	67.76 ± 1.30	64.63–70.83	67.59 ± 1.45	67.51–69.61	68.31 ± 0.87
Pre-anal-fin length (PAFL)	77.71	77.50–82.66	**79.78 ± 1.38^c^**	76.44–81.06	**77.87 ± 1.42^b^**	75.64–79.95	**77.92 ± 1.30^b^**	75.13–80.84	**78.32 ± 1.49^ab^**	79.15–80.96	**80.06 ± 0.69^ac^**
Pectoral-pelvic-fin insertion (PPOL)	32.88	32.05–36.01	33.79 ± 1.08	31.10–34.56	33.15 ± 1.12	31.09–35.23	32.65 ± 1.11	29.39–36.19	32.86 ± 1.69	32.99–35.11	33.68 ± 0.81
Pelvic-anal-fin insertion (PAOL)	24.93	24.93–29.72	27.83 ± 1.44	25.99–29.31	27.40 ± 1.04	26.53–30.34	28.32 ± 1.09	24.09–29.30	27.54 ± 1.26	25.21–29.28	28.01 ± 1.52
Width between mid-pectoral base (BPW)	17.96	15.64–18.23	**17.16 ± 0.68^c^**	17.13–19.80	**18.24 ± 0.84^b^**	18.03–20.68	**19.41 ± 1.06^a^**	16.41–21.72	**19.14 ± 1.37^ab^**	17.01–19.11	18.08 ± 0.82^abc^
Width between mid-pelvic base (BVW)	12.86	11.81–13.73	**12.75 ± 0.56^d^**	12.67–14.37	**13.48 ± 0.46^c^**	13.74–15.97	**14.57 ± 0.76^a^**	12.14–15.80	**13.82 ± 0.83^bc^**	13.24–14.30	**13.70 ± 0.41^abc^**
Upper caudal-lobe length (UCL)	24.32	20.89–26.02	24.30 ± 1.44^abc^	21.15–24.89	**22.82 ± 0.94^b^**	20.93–29.34	24.36 ± 2.06^abc^	22.35–28.53	**25.05 ± 1.74^c^**	22.02–26.70	24.46 ± 1.71^abc^
Lower caudal-lobe length (LCL)	26.64	19.26–27.53	**24.75 ± 2.15^b^**	21.28–27.07	**23.58 ± 1.53^b^**	22.60–29.94	25.51 ± 1.95^ab^	21.72–31.24	**26.70 ± 2.22^a^**	24.19–28.53	25.82 ± 1.78^ab^
**% HL**											
Snout length (SnL)	52.23	47.82–63.77	**55.67 ± 4.37^b^**	53.98–61.11	57.32 ± 1.90 ^ab^	54.84–61.36	57.61 ± 2.08 ^ab^	54.48–61.93	57.65 ± 2.27^ab^	58.70–61.66	**60.20 ± 1.04^a^**
HD	41.75	41.75–61.92	**53.39 ± 4.85^a^**	45.22–54.02	**49.77 ± 2.39^b^**	44.42–50.36	**47.58 ± 1.85^b^**	43.54–51.62	**48.47 ± 2.16^b^**	47.84–52.57	49.97 ± 1.76^ab^
HW	69.14	69.14–97.27	**86.30 ± 8.36^a^**	74.31–81.83	**78.89 ± 2.25^b^**	74.93–82.53	**78.17 ± 2.50 ^b^**	75.72–86.97	**82.06 ± 2.89^b^**	78.23–85.34	**81.80 ± 3.07^ab^**
Eye diameter (ED)	11.93	11.93–20.38	**15.96 ± 2.64^a^**	10.77–14.97	**12.62 ± 1.35^c^**	12.95–17.02	**15.03 ± 1.33^ab^**	12.65–17.88	**15.22 ± 1.29^ab^**	11.63–15.06	**13.50 ± 1.32^b^**
Inter-orbital width (IOW)	33.29	33.29–43.75	**39.04 ± 2.60^a^**	33.83–47.21	**39.42 ± 3.54^a^**	29.42–36.45	**34.15 ± 1.84^b^**	32.00–38.38	**34.80 ± 1.89^b^**	31.03–37.59	**34.80 ± 2.63^b^**
Post-orbital length (POL)	39.26	22.64–39.26	**30.54 ± 5.35^b^**	30.29–38.25	**35.31 ± 1.98^a^**	33.75–39.93	**36.20 ± 2.04^a^**	32.25–40.53	**35.78 ± 1.94^a^**	31.35–35.52	33.61 ± 1.59^ab^
Mouth width (MW)	34.63	24.14–34.63	**27.27 ± 3.16^b^**	31.89–40.61	**36.12 ± 2.53^a^**	22.68–32.65	**28.54 ± 2.66^b^**	23.66–33.98	**27.78 ± 2.32^b^**	30.90–40.11	**36.01 ± 2.99^a^**
**% of caudal peduncle length (CPL)**											
Caudal-peduncle depth (CPD)	71.09	71.09–122.51	**96.11 ± 18.38^a^**	77.12–112.02	89.40 ± 10.17^ab^	69.53–100.17	**83.08 ± 9.44^b^**	71.86–100.14	**84.39 ± 7.20^b^**	83.50–94.32	88.95 ± 3.99^ab^
**% ED**											
Outermost pair of rostral barbels length (OBL)	128.16	78.10–167.14	109.07 ± 24.97^ab^	92.95–159.71	**122.11 ± 22.06^a^**	69.60–105.51	**90.76 ± 10.34^b^**	74.55–118.26	**93.52 ± 12.50^b^**	110.00–145.89	**124.76 ± 12.86^a^**
Maxillary barbel length (MBL)	97.09	49.75–118.18	82.43 ± 21.24^ab^	63.75–114.49	**84.90 ± 16.14^a^**	51.54–83.67	**67.80 ± 9.77^b^**	53.57–86.67	71.89 ± 9.34 ^ab^	77.37–94.69	82.77 ± 7.04 ^ab^
Lower lip short barbel length (LBL)	32.04	24.34–51.98	**34.39 ± 6.85^a^**	16.10–41.73	**26.75 ± 6.16^b^**	21.13–38.87	30.07 ± 5.95^ab^	21.59–45.13	32.28 ± 5.73^ab^	26.84–42.66	**36.16 ± 5.63^a^**
**%** POW											
SnL	133.04	133.04–233.97	**187.08 ± 32.22^a^**	143.37–194.32	**162.90 ± 12.41^b^**	140.92–179.01	**159.72 ± 12.37^b^**	137.17–181.73	**161.72 ± 13.07^b^**	167.50–193.75	179.56 ± 10.63ab
**Meristic counts**											
Dorsal-fin rays	iii,8	iii,8		iii, 8–9		iii, 8		iii, 8		iii, 8	
Pectoral-fin rays	i, 14	i, 14–15		i, 14–15		i, 15		i, 15		i, 16	
Pelvic-fin rays	i,8	i,8		i, 7–8		i, 8–9		i, 8		i, 8	
Anal-fin rays	ii,5	ii, 5		ii, 5		ii, 5		ii, 5		ii, 5	
Lateral-line scales	87	**72–88^a^**		73–86^ab^		**76–88^a^**		**65–83^b^**		**78–90^a^**	

Note: Superscript letters indicate statistically significant differences (*p* < 0.05) based on one-way ANOVA with post-hoc comparisons.

Body anteriorly subcylindrical, ventral profile flat from head to abdomen; dorsal profile gently rising from snout tip to dorsal-fin origin, then sloping to caudal-fin base; posterior body laterally compressed behind pelvic-fin base. Head slightly depressed, widest posteriorly; width greater than depth; 19.9–27.1% SL. Snout rounded, blunt; length 1.33–2.34× postorbital length. Mouth small, inferior, arched. Lips fleshy; upper lip broad, papillae absent; lower lip with one pair of short pointed posterior barbels and one pair of anterior lobe-like protrusions. Maxillary barbels one pair at mouth corners, each with basal pad bearing variably distinct pointed wart-like projection. Upper and lower lips connected at mouth corners by skin flaps and maxillary barbels; upper jaw concealed by upper lip.

Rostral groove present between upper lip and snout tip, bearing four primary barbels with bases in groove, alternating with tertiary pairs. Rostral fold anterior to groove, margin bearing six tertiary barbels arranged in three pairs; inner surface with three secondary barbels between each tertiary pair, bases on fold. Anterior and posterior nostrils adjacent, small, nasal flaps well developed. Eyes round; diameter 12.0–20.4% HL. Gill openings large, extending onto ventral surface of head.

Scales minute; head scaleless. Body scaled dorsally and ventrally to posterior of pelvic-fin origin. Ventrolateral scaling from pelvic-fin origin to pectoral axilla; scales medially embedded or absent; embedded scales first visible anterior to pectoral-fin axilla. Lateral line complete; 72–88 scales.

Dorsal-fin origin above and slightly anterior to pelvic-fin origin; insertion at or slightly anterior to mid-distance between snout tip and caudal-fin base; length 21.8–26.4% SL. Pectoral fin laterally expanded, margin curved. Pelvic fin laterally expanded, with a dorsal fleshy flap at base; tip surpassing anus. Anal fin reaching or slightly surpassing caudal-fin base when adpressed; length 17.0–19.7% SL. Caudal peduncle laterally compressed; depth 0.71–1.23× length. Caudal fin forked when compressed, slightly emarginate when fully expanded; lower lobe slightly longer.

##### Colouration in preserved specimens.

Dorsum pale yellow, patterned brown to black; venter creamy white. Dorsal surface of head with dark vermiculate markings. Predorsal region typically with one continuous longitudinal rectangular dark blotch, margins even to weakly undulate, 2–3 small light brown saddle spots occasionally connecting to lateral pale stripes. Post-dorsal region with 2–4 saddle-shaped patches separated by pale interspaces, connected to pale lateral stripes. Dorsal surface of precaudal base with one black spot. After long-term preservation in low-concentration alcohol, yellowish areas intensified

Flank with broad, dark longitudinal band along lateral line, 2–5 scales wide, margins even to weakly undulate; a straight pale longitudinal band, 1–3 scales wide, between lateral dark band and dorsal pigmentation, usually continuous to posterior margin of head, occasionally interrupted near pectoral region. Transition zone between flank and venter variably pale or bearing narrow continuous to interrupted dark stripe. Bases of paired fins dorsally with small black spot.

Fin membranes hyaline white. Paired fins with 0–2 broad dark bars perpendicular to rays, dorsal fin with 2–3 bars, anal fin with one bar, caudal fin with 3–4 bars; lower half of caudal-fin base with a narrow, transverse, elliptical, black spot.

##### Colouration in life.

Bright spots on body at dorsal base of paired fins, anterior and posterior to dorsal-fin base, and on upper half of caudal-fin base (Fig. 4).

##### Colouration of juvenile specimens.

Predorsal region with continuous dark longitudinal stripe enclosing 1–2 pale, central, rectangular areas; postdorsal region with 2–3 fragmented saddle patches. Lateral line with straight, continuous, dark stripe extending through eye to snout. Fin markings faint, except on caudal fin.

##### Etymology.

The species name honours Wen-Qiao Tang, a senior Chinese ichthyology researcher, and is derived from the latinized Chinese spelling of his family name, in recognition of his contributions to the field. We propose “Wén Qiáo Yīng Kŏu Qiū” (文乔缨口鳅) as its Chinese common name.

##### Distribution and habitat.

This species is endemic to the Jiulong River (九龙江) system, which flows independently into the sea in southeastern China. This species inhabits streams with gravel or pebbly substrates and has a carnivorous-leaning omnivorous feeding habit.

##### Molecular analysis.

We conducted phylogenetic analysis using 53 sequences. After alignment and trimming, a 1140-bp sequence was obtained, containing 788 conserved sites, 352 variable sites, 110 singleton sites, and 242 parsimony-informative sites. The average nucleotide frequencies were A = 25.7%, T = 27.9%, C = 30.3%, and G = 16.1%, showing an A–T bias (53.6%).

**Figure 5.**
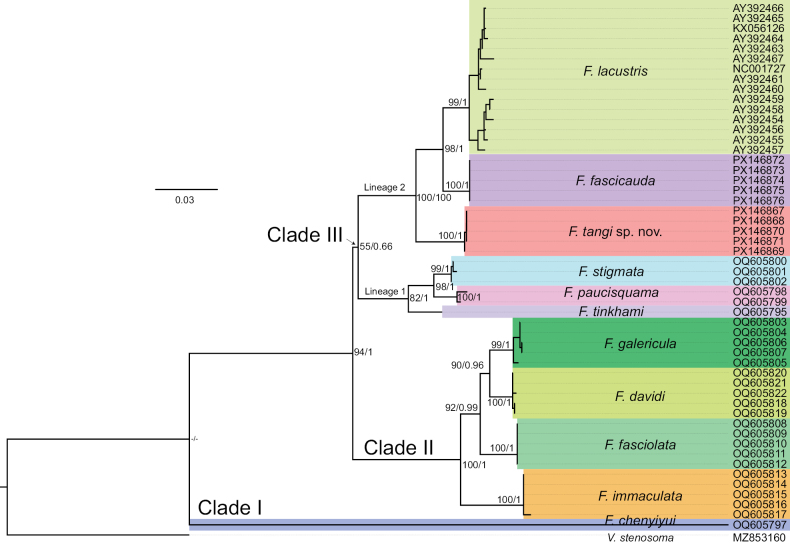
Bayesian-inference tree based on mitochondrial *cytb* gene sequences from 11 species of *Formosania*. Maximum-likelihood and Bayesian-inference analyses yielded congruent topologies. ML bootstrap / BI posterior probability is displayed at superspecific nodes; a dash (“–”) indicates that the value is not applicable.

Both maximum-likelihood and Bayesian-inference analyses produced fully congruent tree topologies. All species formed well-supported monophyletic groups (ML bootstrap ≥ 99, BI posterior probability = 1). The genus *Formosania* was divided into three major clades: *F. chenyiyui* alone constituted clade I, which was recovered as the sister group to all other species. Clade II comprised all species characterized by a single row of rostral barbels, namely *F. davidi*, *F. fasciolata*, *F. galericula*, and *F. immaculata*. The remaining species fell into clade III, which was further resolved into two distinct lineages. Lineage 1 included *F. stigmata*, *F. paucisquama*, and *F. tinkhami*. Lineage 2 consisted of *F. lacustris*, which also encompasses *F. gilberti* under the taxonomic framework of Yeh et al. (2024), *F. fascicauda*, and the new species *F. tangi* sp. nov., positioned at the base of this lineage. All nodes were strongly supported except for the root of clade III, which received moderate support (ML = 55%, BI = 0.66) (Fig. 5).

Genetic distances among the 11 *Formosania* species were calculated using the Kimura 2-parameter (K2P) model. The smallest genetic distance to *F. tangi* sp. nov. was observed in *F. fascicauda* (4.2%). Notably, this interspecific divergence exceeded that between many other congeneric species pairs, such as *F. fascicauda* and *F. lacustris* (2.93%), among *F. tinkhami*, *F. stigmata*, and *F. paucisquama* (2.12–3.48%), and among *F. davidi*, *F. fasciolata*, and *F. galericula* (2.40–3.05%) (Table 3).

**Table 3.** Inter-specific mean mitochondrial cyt *b* genetic distances within the genus *Formosania* in Kimura 2-parameter genetic distance analysis (%).

	1	2	3	4	5	6	7	8	9	10
1. *F. tangi* sp. nov.										
2. *F. lacustris*	4.67									
3. *F. chenyiyui*	17.91	17.19								
4. *F. tinkhami*	6.88	7.41	16.38							
5. *F. paucisquama*	7.12	7.72	16.81	3.48						
6. *F. stigmata*	7.02	7.46	16.18	3.19	2.12					
7. *F. fascicauda*	4.16	2.93	15.97	7.00	7.84	7.33				
8. *F. immaculata*	9.69	9.55	18.69	8.60	8.79	9.08	8.68			
9. *F. galericula*	9.28	9.12	17.90	9.05	8.87	8.97	8.53	4.80		
10. *F. fasciolata*	9.43	9.54	18.23	9.05	8.88	9.02	8.51	4.94	3.05	
11. *F. davidi*	9.26	9.22	18.64	8.78	9.06	8.87	8.35	4.73	2.40	2.96

##### Morphometric traits.

**Figure 6.**
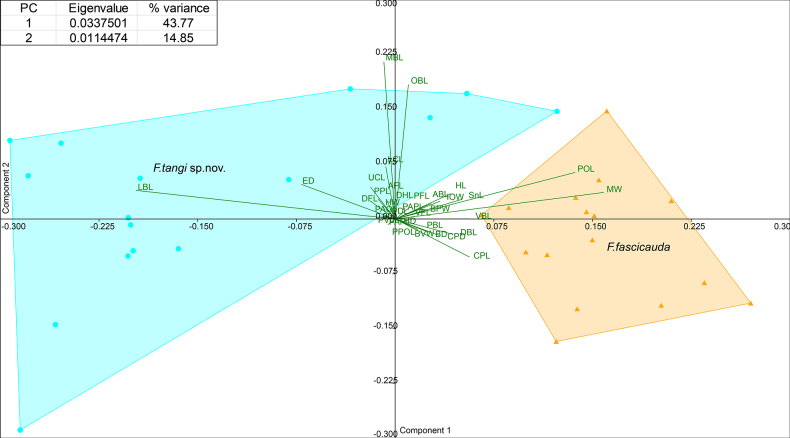
Principal component analysis (PCA) ordination plot of morphometric traits for *Formosania tangi* sp. nov. and *F. fascicauda* based on PC1 and PC2.

The principal component analysis (PCA) of morphometric traits clearly separated *F. tangi* sp. nov. and *F. fascicauda* along the first two principal components. PC1 and PC2 accounted for 47.77% and 14.85% of total morphological variation, respectively, together defining the major axes of divergence (Fig. 6). The morphospace occupied by each species, represented by blue (*F. tangi*) and orange (*F. fascicauda*) polygons, showed clear separation, primarily along PC1. Specimens of *F. tangi* clustered in quadrants 1–3, mostly left of the y-axis, and were characterized by positive loadings on MBL, OBL, and LCL, and negative loadings on LBL and ED. In contrast, *F. fascicauda* grouped in quadrants 1 and 4, influenced mainly by positive loadings on POL, MW, and CPL.

Although one-way ANOVA identified significant differences in certain morphometric ratios among the double-row barbel species, all variables exhibited broad interspecific overlap. Thus, morphometric traits offer limited diagnostic utility for distinguishing these species (Table 2).

## Discussion

### Caudal fin

**Figure 7.**
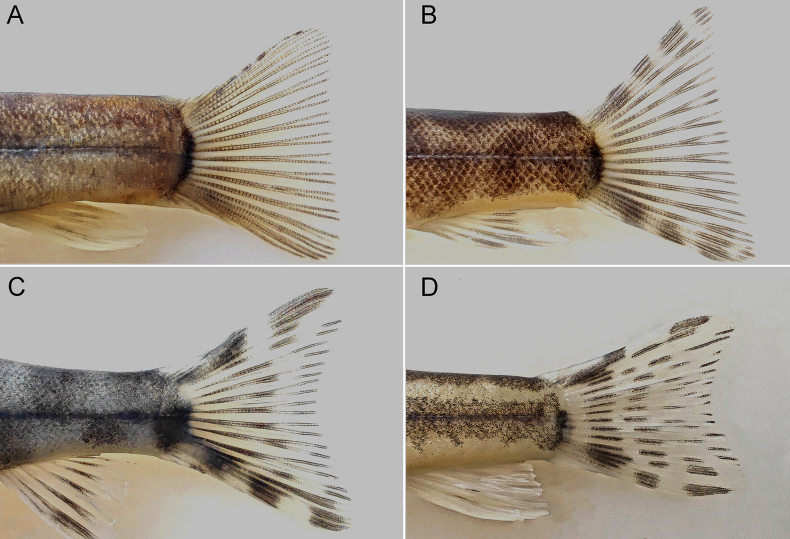
Caudal fin morphotypes in the genus *Formosania*. **A**. Plain subtruncate (*F. davidi*); **B**. Patterned subtruncate (*F. fascicauda*); **C, D**. Patterned emarginate (**C**. *F. stigmata*; **D**. *F. tangi* sp. nov.).

The fully expanded caudal fin within the genus *Formosania* exhibits three distinct morphological patterns: a plain subtruncate type, a patterned subtruncate type, and a patterned emarginate type (Fig. 7). The plain subtruncate type, which is characterized by a nearly straight to slightly oblique posterior margin and the absence of distinct dark stripes on the caudal-fin rays, is found exclusively in the *F. davidi* species group (clade I in the phylogeny; Fig. 7A). The patterned subtruncate type, featuring a distinctly oblique posterior margin and 3–4 prominent dark stripes on the caudal-fin rays, is unique to *F. fascicauda* (Fig. 7B). All remaining species, including *F. tangi* sp. nov., display the patterned emarginate type, which is defined by a slightly concave posterior margin and the presence of 3–4 prominent dark stripes on the caudal fin rays (Fig. 7C, D).

We emphasize that the preceding morphological comparisons pertain specifically to the fully expanded state of the caudal fin. In an unexpanded condition, all three types exhibit a central concavity of varying extent. Moreover, while the first two types may not present a perfectly obliquely truncate posterior margin, their concavity is markedly shallower than that characteristic of the patterned emarginate type.

### Flank markings

**Figure 8.**
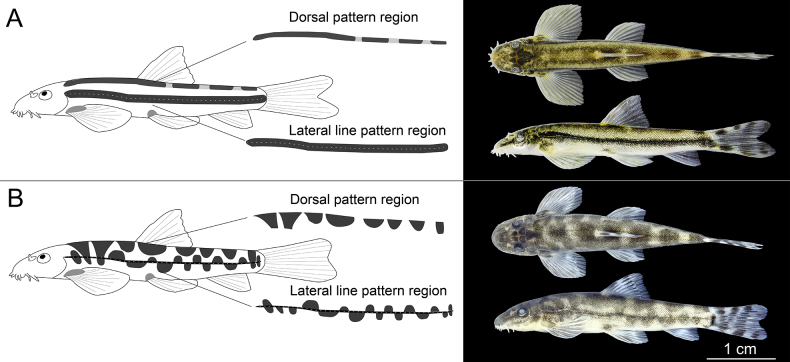
Pattern differences between *Formosania tangi* sp. nov. (**A**) and *F. fascicauda* (**B**), caudal fin not fully expanded.

*Formosania tangi* sp. nov. is readily distinguished from *F. fascicauda* by its distinctive flank markings, with differences evident from juvenile to adult stages (earlier developmental stages were not obtained). *Formosania tangi* displays a straight, dark, mid-lateral stripe along the lateral line, which is consistently broader than in *F. fascicauda*. A dark dorsal stripe runs mid-dorsally in juveniles; the section behind the dorsal fin gradually forms regular, saddle-like blotches during growth. In contrast, *F. fascicauda* shows a thin, dark mid-lateral line with alternating dorsal and ventral blotches that enlarge and become irregular with age. Along the dorsum, it exhibits 5–7 saddle-shaped blotches, regular in juveniles but turning irregular and laterally extended in adults. Similar marking patterns occur in other species with two rows of barbels such as *F. stigmata* and *F. paucisquama* (Fig. 8)

### Resolution of a long-term taxonomic misidentification

Chen’s (1980) report of *F. fascicauda* from the Mulanxi and Jiulongjiang rivers constituted the most accurate interpretation possible given the material and methods available at the time. Subsequently, its incorporation into Fauna Sinica (Chen and Tang 2000) conferred institutional authority on this identification, which shaped the scientific understanding of these populations for decades.

We now recognize this interpretation as an understandable misassignment, stemming from the absence of topotypic *F. fascicauda* for direct comparison and the inherently subtle morphological differentiation among many *Formosania* populations in the coastal basins of Fujian. In this context, *F. tangi* sp. nov. presents a clear exception with its distinctive pigment pattern, whereas populations in the Jinjiang and Mulanxi rivers remain morphologically similar to true *F. fascicauda* – a continuity that reasonably led earlier workers to presume intraspecific variation.

**Figure 9.**
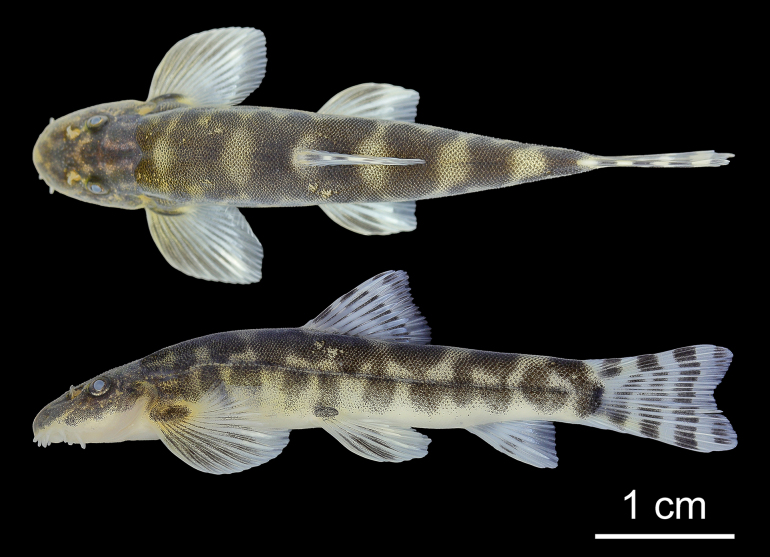
Dorsal and ventral views of *Formosania fascicauda*. (SHOU20251010101, from Longjiang River, the type locality, in Fuqing City, Fujian Province), caudal fin not fully expanded.

Our integrated analysis, based on topotypic specimens (Fig. 9) and molecular data, confirms that *F. tangi* is morphologically diagnosable and phylogenetically distinct. Under the current taxonomic framework that retains *F. fascicauda*, *F. lacustris*, and *F. gilberti* as valid species, *F. tangi* does not form a monophyletic group with any of these recognized taxa. These consistent lines of evidence firmly support the recognition of *F. tangi* as a distinct species.

The present analysis conclusively establishes that *F. tangi* and *F. fascicauda* can be reliably distinguished based on external morphology. The most prominent diagnostic trait is the lateral body pattern. In *F. tangi*, this pattern consists of a broad, dark, longitudinal band accompanying the lateral line, with margins that are weakly undulating to nearly straight. This band is separated from the darker dorsal surfaces by a generally continuous, straight, pale stripe (Fig. 8A), a configuration that is highly distinctive in *Formosania*. In contrast, *F. fascicauda* exhibits a thin, black longitudinal line composed of alternating patches, with the pale stripe being intermittent, wavy, or entirely absent (Fig. 8B)—a pattern more similar to those observed in congeners such as *F. stigmata* and *F. paucisquama*. Furthermore, caudal fin morphology serves as a reliable secondary diagnostic character. When fully expanded, the caudal fin of *F. tangi* is distinctly emarginate. This condition contrasts clearly with that of *F. fascicauda*, in which the fully expanded fin is typically subtruncate. It is noteworthy that even in the occasional *F. fascicauda* specimen where the fully expanded caudal fin is not perfectly truncate and shows slight incurvation, it never exhibits the pronounced emargination characteristic of *F. tangi*. Collectively, these consistent and observable morphological differences robustly support the distinction between the two species.

### Diagnostic key to species of *Formosania*

**Table:** 

1	Tertiary rostral barbels occasionally papilliform; barbels and papillae 12–15; dorsum with broad longitudinal dark band, incurved at dorsal-fin axil	**(Hanjiang River) *F. chenyiyui***
–	Rostral barbels well developed; barbels 13; dorsum with saddle-shaped blotches or reticulated pattern	**2**
2	Rostral barbels in single row, continuous with rostral fold; median caudal-fin rays unpigmented	**3**
–	Rostral barbels in two rows, posterior row situated along rostral groove; caudal-fin with 2–4 dark transverse stripes	**6**
3	Body unmarked; snout about 1.2 times postorbital length	**(Oujiang River) *F. immaculata***
–	Body marked; snout subequal to postorbital length	**4**
4	Abdominal scaleless area restricted anterior to midpoint of pectoral-fin base	**(Feiyunjiang, Oujiang and Aojiang rivers) *F. fasciolata***
–	Abdominal scaleless area extending slightly beyond pectoral-fin axilla	**5**
5	Longest rostral barbel about 1.5 times eye diameter; dorsum with 7–9 saddle-shaped bands	**(Minjiang and Xinjiang rivers) *F. davidi***
–	Rostral barbels subequal to eye diameter; dorsum with reticulated pattern	**(Oujiang River) *F. galericula***
6	Lateral line bordered by broad dark longitudinal band, margins weakly undulate to nearly straight; pale mid-dorsal stripe continuous and straight	**(Jiulongjiang River) *F. tangi* sp. nov**.
–	Lateral line with a thin black longitudinal line composed of alternating patches, or small alternating dark dots, or a continuous narrow line; pale mid-dorsal stripe intermittent, wavy, or absent	**7**
7	Fully expanded caudal-fin oblique subtruncate; postdorsal flank with a pale intermittent wavy stripe	**(Longjiang and Yuxi rivers) *F. fascicauda***
–	Fully expanded caudal fin emarginate; pale stripe absent	**8**
8	Lateral line with a thin black longitudinal line with alternating patches	**9**
–	Lateral line with small alternating dark dots, or a black line	**10**
9	Lateral line scales 86–92	**(Minjiang and Jiaoxi rivers) *F. stigmata***
–	Lateral line scales 76–83	**(Rongjiang, Lianjiang, Hanjiang Rivers) *F. paucisquama***
10	Flanks unblotched	**(Jhuoshui, Zhengwen and Nei Shuong rivers, Taiwan) *F. lacustre***
–	Flanks with interrupted dark short streaks or small patches along dorsal and ventral margins, or vermiculate	**11**
11	Flanks vermiculate	**(Tamshui, Beihuang, Shuang and Masu Rivers, Taiwan) *F. gilberti***
–	Flanks with interrupted dark short streaks or small patches along dorsal and ventral margins	**(Pearl River) *F. tinkhami***

### Comparative materials

*Formosania tangi* sp. nov. • SHOU20240704603-605, SHOU20251010601-602, 5 specimens, 56.84–73.69 mm SL; China, Fujian Province, Sanming City, Jiulongjiang River. • SHOU202201083-093, 11 specimens, 50.53–81.06 mm SL; China, Fujian Province, Zhangzhou City, Jiulongjiang River.

*Formosania fascicauda* • SHOU20241019001-005, SHOU20241019007-008, SHOU20241019013-014, SHOU20241019016-019, SHOU20241019021-022, 15 specimens, 53.98–83.30 mm SL; China, Fujian Province, Fuqing City, Yuxi River. • SHOU20241122001, 1 specimen, 74.24 mm SL; China, Fujian Province, Fuqing City, Longjiang River.

*Formosania stigmata* • SHOU2021060126-135, 10 specimens, 50.04–75.85 mm SL; China, Zhejiang Province, Lishui City, Jiaoxi River. • SHOU2021060180-183, 4 specimens, 50.98–85.47 mm SL; China, Zhejiang Province, Lishui City, Minjiang River.

*Formosania paucisquama* • SHOU202311001-013, 13 specimens, 49.62–65.76 mm SL; China, Guangdong Province, Meizhou City, Hanjiang River. • SHOU202101001-005, 5 specimens, 52.26–64.03 mm SL; China, Guangdong Province, Jieyang City, Rongjiang River. • SHOU202110011, 1 specimen, 49.04 mm SL; China, Guangdong Province, Jieyang City, Lianjiang River.

*Formosania tinkhami* • SHOU20231108001-006, 6 specimens, 51.98–80.29 mm SL; China, Guangdong Province, Huizhou City, Pearl River.

## Supplementary Material

XML Treatment for Formosania tangi

## Additional information

### Use of AI

No use of AI was reported.

### Author ORCIDs

Yang Chen https://orcid.org/0009-0009-9923-917X

Jia-Jun Zhou https://orcid.org/0000-0003-1038-1540

Jing-Chen Chen https://orcid.org/0000-0001-9164-6920

Jin-Quan Yang https://orcid.org/0000-0003-0387-1824

### Data availability

All of the data that support the findings of this study are available in the main text.

## References

[B1] Burland TG (2000) DNASTAR’s Lasergene sequence analysis software. Methods in Molecular Biology (Clifton, N.J.) 132: 71–91. 10.1385/1-59259-192-2:71 10547832

[B2] Chen YY (1980) Systematic studies on the fishes of the family Homalopteridae of China II. Classification of the fishes of the subfamily Gastromyzoninae. Shui Sheng Sheng Wu Hsueh Bao 7(1): 95–120. 10.3724/issn1000-3207-1980-1-95-c [In Chinese]

[B3] Chen YY, Tang WQ (2000) Homalopteridae. In: Le PQ (Ed.) Fauna Sinica, Osteichthyes, Cypriniformes III. Science Press, Beijing, 438–567. [In Chinese]

[B4] Chen JC, Li JJ, Tang WQ, Pu XR, Lei HT (2024) Taxonomic resolution of the hillstream suck-loach *Beaufortia pingi* species group (Cypriniformes, Gastromyzontidae) and two new species from southwest China – *Beaufortia granulopinna* and *Beaufortia viridis*. Zoosystematics and Evolution 100(3): 941–963. 10.3897/zse.100.124370

[B5] Fang PW (1934) Study on the crossostomoid fishes of China. Sinensia 6(1): 44–97.

[B6] Fricke R, Eschmeyer WN, Van der Laan R [Eds] (2026) Eschmeyer’s catalog of fishes: genera, species, references. http://researcharchive.calacademy.org/research/ichthyology/catalog/fishcatmain.asp

[B7] Kalyaanamoorthy S, Minh BQ, Wong TKF, von Haeseler A, Jermiin LS (2017) ModelFinder: Fast model selection for accurate phylogenetic estimates. Nature Methods 14(6): 587–589. 10.1038/nmeth.4285 PMC5453245 28481363

[B8] Katoh K, Standley DM (2013) MAFFT multiple sequence alignment software version 7: Improvements in performance and usability. Molecular Biology and Evolution 30(4): 772–780. 10.1093/molbev/mst010 PMC3603318 23329690

[B9] Nguyen LT, Schmidt HA, von Haeseler A, Minh BQ (2015) IQ-TREE: A fast and effective stochastic algorithm for estimating maximum-likelihood phylogenies. Molecular Biology and Evolution 32(1): 268–274. 10.1093/molbev/msu300 PMC4271533 25371430

[B10] Nichols JT (1926) Some Chinese fresh-water fishes. XVIII. New species in recent and earlier Fukien collections. American Museum Novitates 224: 1–7.

[B11] Novák J, Hanel L, Rícan O (2006) *Formosania*: A replacement name for *Crossostoma* Sauvage, 1878 (Teleostei), a junior homonym of *Crossostoma* Morris & Lycett, 1851 (Gastropoda). Cybium 30(1): 92. 10.26028/cybium/2006-301-016

[B12] Oshima M (1919) Contributions to the study of the fresh water fishes of the island of Formosa. Annals of the Carnegie Museum 12(2–4): 169–328. 10.5962/p.34608

[B13] Rambaut A (2018) FigTree v1.4.4. http://tree.bio.ed.ac.uk/software/figtree/

[B14] Ronquist F, Teslenko M, van der Mark P, Ayres DL, Darling A, Höhna S, Larget B, Liu L, Suchard MA, Huelsenbeck JP (2012) MrBayes 3.2: Efficient Bayesian phylogenetic inference and model choice across a large model space. Systematic Biology 61(3): 539–542. 10.1093/sysbio/sys029 PMC3329765 22357727

[B15] Sauvage HE (1878) Note sur quelques Cyprinidae et Cobitidae d’espèces inédites, provenant des eaux douces de la Chine. Bulletin de la Société Philomathique de Paris (7^th^ Série) 2: 86–90.

[B16] Sun W, Zhou JJ, Yang JQ (2023) *Formosania immaculata*, a new species of hillstream loach (Teleostei, Cypriniformes, Gastromyzontidae) from the Ou-Jiang River, southeast China. ZooKeys 1182: 207–221. 10.3897/zookeys.1182.104240 PMC10594117 37881412

[B17] Sun ZX, Li XY, Li XJ, Hao JY, Sheng D, Zhao YH (2025) *Cobitis beijingensis*, a new spined loach from northern China (Cypriniformes, Cobitidae). Zoosystematics and Evolution 101(1): 55–67. 10.3897/zse.101.137363

[B18] Tamura K, Stecher G, Kumar S (2021) MEGA11: Molecular Evolutionary Genetics Analysis version 11. Molecular Biology and Evolution 38(7): 3022–3027. 10.1093/molbev/msab120 PMC8233496 33892491

[B19] Yeh MF, Li HE, Han CC, Chen IS (2024) Redescription and validity of *Formosania gilberti* Oshima, 1919 (Cypriniformes: Balitoridae), an endemic hillstream loach from Taiwan. Zootaxa 5550(1): 287–304. 10.11646/zootaxa.5550.1.29 40173642

[B20] Zhang CG, Zhao YH (2016) Species Diversity and Distribution of Inland Fishes in China. Science Press, Beijing, 296 pp. [In Chinese]

[B21] Zhang D, Gao F, Jakovlić I, Zou H, Zhang J, Li WX, Wang GT (2020) PhyloSuite: An integrated and scalable desktop platform for stream lined molecular sequence data management and evolutionary phylogenetics studies. Molecular Ecology Resources 20(1): 348–355. 10.1111/1755-0998.13096 31599058

